# Correlation between physical activity, eating behavior and obesity among Sudanese medical students Sudan

**DOI:** 10.1186/s40795-019-0271-1

**Published:** 2019-02-06

**Authors:** Marwa Mohammed Yousif, Lamis AbdelGadir Kaddam, Humeda Suekit Humeda

**Affiliations:** 1 Department of Physiology Faculty of Medicine, Al-Neelain University, P.O. Box: 11121, 12702 Khartoum, Sudan; 2 Department of Physiology Faculty of Medicine, International University of Africa, Khartoum, Sudan

**Keywords:** Obesity, Physical activity, Medical students, Eating behavior

## Abstract

**Background:**

Obesity has emerged as a major health problem. Prevalence is increasing hugely. Various etiological factors had been identified as potential causes of obesity. There is an increasing need to study different determinants of obesity especially the physical activity and eating habits. Future doctors considered as role models in community. Doctor wellbeing’s does not just affect them it has robust impact on their surroundings. More knowledge about determinants of obesity among medical students may shed light concerning obesity prevention and control. Therefore, the aim of this study was to determine the relationships between physical activity, eating patterns, and obesity among medical students.

**Methods:**

This was a cross sectional study conducted among 216 medical students at Al-Neelain University selected by stratified random sampling. Data were collected by self-administered questionnaire, which included background data. International physical activity questionnaire (IPAQ) was used to determine physical activity level and the three factors eating questionnaire Revised 18 (TFEQ-r18) was used to determine eating behavior. Also, anthropometric measurements were performed for each participant. Data were analyzed using SPSS version 23 program. Descriptive data were presented as means ± SD and percentages. The relationships between BMI and physical activity levels and between BMI and eating pattern were analysed using non parametric test. *P* value < 0.05 was considered significant. .

**Results:**

The prevalence of obesity among students was 6.5% and overweight was 22.2%. The study showed that 44.9% of medical students had low activity level while 32% of students had moderate activity level and 23.1% had high physical activity level. There was no significant relationship between physical activity and body mass index (BMI) in this study. The common eating pattern among students was controlled eating (45.8%). There was significant relationship between eating behaviors and BMI (*P* = 0.01).

**Conclusion:**

The study revealed eating habits has stronger impact on BMI than physical activity. Disturbing figures regarding overweight and low physical activity among medical students, identified in this study, encourages implementation of health programs. Emphasize on importance and benefits of physical activity and eating habits in medical curricula.

## Background

As life changes and becomes more developed there are a lot of health problems and diseases immerges. One of these major health problems is obesity. Nearly 20–40% of adult and 10–20% of children worldwide are obese [1]. The prevalence of overweight and obesity in Zambia was 24.7% [2]. And in Algeria, prevalence of overweight and obesity among adults was respectively 32.5 and 30.9% [3]. In Ethiopia, prevalence of overweight and/or obesity was found to be 9.4% [4]. While in Nigeria overweight and obesity among civil servants in Lagos, was70.7% [5]. In Sudan the prevalence of obesity in 2016 is 8.6% based on WHO statistics [6]. Overweight and obesity are defined as abnormal or excessive fat accumulation that may impair health [7]. Body mass index (BMI) is used to classify overweight and obesity in adults. It is person’s weight in kilograms divided by the square of his height in meters (kg/m^2^). Calculated BMI greater than or equal to 25 is considered overweight; while obesity is considered when BMI is greater than or equal to 30 [7].

Various epidemiological determinants have been identified as possible causes of obesity. Including dietary patterns, dietary habits, physical activities, alcohol consumption, stress, and family history of any chronic health problem like obesity, diabetes, hypertension, etc. [1]. The level of physical activity is reduced in developing countries and sedentary behaviors have risen, which may contribute to increased incidence of obesity and other chronic diseases like diabetes and hypertension [8] .For 2030 and beyond, obesity is likely to be higher in low income countries [9].

Prospective observational population studies of adults, from the last 20 years,with physical activity measured at baseline, were few and disclosed inconsistent results with regard to the effect of physical activity on body weight and development of obesity [10]. In Sudan, obesity problem is associated with unhealthy dietary habits. It has been found that less than half of Sudanese students eat vegetables more than 3 days per week [8]. Although many studies had been conducted to investigate the different causes of obesity among adults, very few attempts were made among medical students in Sudan. In addition, no previous studies were conducted to assess the physical activity and eating behavior using validated and well recognized methods.

Since medical student are the future doctors and role models in our community. They will have heavy stressful work in future and they need to be healthy and to keep themselves away from risk factors of diseases like obesity. Doctors’ behavior doesn’t just impact them and their families it had an impact on all community and country recourses.

Therefore, the aim of this study was to determine the relationship between physical activity, eating behavior, and obesity among medical students at Al-Neelain University.

## Methods

The study was a cross sectional study conducted at the Faculty of Medicine Al-Neelain University. The sample size was 216 students calculated using the formula: n = N/1 + N (D) ^2^ and the participants were selected by stratified random sampling from official list of medical students from first to fifth year. Only Sudanese students were included in this study. Students diagnosed with renal disease, endocrine disease, or chronic illness were excluded.

Background data including age, gender & study level were collected using self- administered questionnaire.

### IPAQ

The International Physical Activity Questionnaire (IPAQ) was developed into short and long versions. These questionnaires could be administered by telephone interview or self-administration. There are two different reference periods under investigation, either the “last 7 d” or a “usual week” [11] .

In this study, we utilized the short form of international physical activity questionnaire (SF-IPAQ) to determine the level of physical activity. It contains 7 questions covering all types of physical activity [12]. IPAQ assesses physical activity undertaken across a comprehensive set of domains including leisure time, domestic and gardening (yard) activities, work-related and transport-related activity. Only the physical activity lasting longer than 10 min was estimated, without rest breaks and within the last 7 days [12]. Physical activities are classified into three categories: vigorous, moderate and walking. Vigorous activity is the physical activity that causes large increases in breathing and or heart rate like carrying or lifting heavy loads, digging, running, football or construction work. Moderate physical activity causes small increases in breathing and or heart rate such as carrying light loads,cycling, swimming and volleyball [12]. All types of walking are included in walking category. For each specific type of activity frequency (measured in days per week) and duration (time per day) are recorded. Physical activities were calculated in Metabolic Equivalent (MET). MET is the ratio of a person’s working metabolic rate relative to the resting metabolic rate [12] . One MET is defined as the energy cost of sitting quietly, and is equivalent to a caloric consumption of 1 kcal/kg/hour. Vigorous activity was given 8 METs, moderate 4 METs and walking 3.3 METs [12]. For each activity the score was calculated by multiplying its METs *frequency (days per week)* duration (time per day in minutes). Therefore, when calculating a person’s overall total physical activity using IPAQ: Total physical activity MET-minutes/week = sum of Total (Walking + Moderate + Vigorous) MET minutes/ week scores [12]. Using these values, there are three levels of physical activity. High physical activity: The two criteria for classification as ‘high’ are: vigorous-intensity activity on at least 3 days achieving a minimum Total physical activity of at least 1500 MET-minutes/week OR 7 or more days of any combination of walking, moderate-intensity or vigorous-intensity activities achieving a minimum total physical activity of at least 3000 MET-minutes/week. Moderate physical activity: The pattern of activity to be classified as ‘moderate’ is either of the following criteria: 3 or more days of vigorous-intensity activity of at least 20 min per day OR 5 or more days of moderate-intensity activity and/or walking of at least 30 min per day OR 5 or more days of any combination of walking, moderate-intensity or vigorous intensity activities achieving a minimum Total physical activity of at least 600 MET-minutes/week [12]. Low physical activity: Those individuals who not meet criteria for high or moderate are considered to have a ‘low’ physical activity level [12].

### TFEQ-R18

The Three Factor Eating Questionnaire Revised 18(TFEQ-R18) consists of three scales that classify subjects to cognitive restraint, emotional eating and uncontrolled eating. It was revised to contain only 21 items from the longer version with 51 items and then again to 18 items for the TFEQ-R18. Although the TFEQ-R18 scales were derived in obese subjects, factor analysis of the TFEQ-R21 conducted in an adult sample indicates that the instrument is valid also in non-obese individuals and has been validated in the general population, as well [13].

(TFEQ-R18) was used to determine eating behaviour of the participants. It’s consists of 18 items on a 4-point response scale (definitely true/mostly true/ mostly false/definitely false).The questionnaire refers to current dietary practice and measures 3 different aspects of eating behaviour: restrained eating (conscious restriction of food intake in order to control body weight or to promote weight loss), uncontrolled eating (tendency to eat more than usual due to a loss of control over intake accompanied by subjective feelings of hunger), and emotional eating (inability to resist emotional cues). Conscious restriction (CR) consists of 6 questions, emotional eating (EE) has 3 questions and uncontrolled eating (UE) has 9 questions [13].

The three questionnaires were pre tested in a sample of 5 students-not included in the study- to assess its ease and clarity of items.

**Anthropometric measurements** were measured using equipment of tested design and calibrated at frequent intervals. Body weight was measured by an Avery beam weighting scale. Adult was weighted in minimal clothing to the nearest 0.1 kg. Standing height was measured, without shoes, to the nearest 0.5 cm using stadiometer (SECA). Body mass index was calculated using this equation: BMI = weight (kg) / Height^2^ (m)^.^ Grading of BMI was done according to WHO grading (normal values: 18.5–24.9 kg/m^2^, below 18.5 are underweight, individuals with BMI ranging from 25 to 29.9 are overweight BMI, above 30 are labeled obese and those with BMI more than 35 are morbid obese) [3].

#### Statistical analysis

Data were analysed using SPSS version 23, Descriptive data were presented as means and standard deviations of means or percentages. Statistical tests used were One- Sample Kolmogorov-Smirnov test to assess if the variables were normally distributed, Independent t-test was used to compare the means among normally distributed variables and Mann Whitney U test among not normally distributed variables. Correlation analysis was used for assessing associations between study variables.Chi-square test with 95% confidence level was used to determine the relationship between the gender and physical activity and between gender and obesity. The relationship between BMI and eating behaviour and between BMI and physical activity level were analysed using non-parametric tests.*P* value less than 0.05 was considered significant.

## Result

The total number of subjects who participated in this study was 216 students, 42% of them were males, and 58% were females. Their ages ranged between 18 to 25 years and the average was 19.99 ± 1.86 years. The participants were from first to fifth level. (Table 1).

**Table 1:** Background characteristics of participants (*n* = 216)

Characteristics	Frequency	%
Gender		
Female	126	58.0
Male	90	42.0
Total	216	100.0
Study level		
1	52	24.0
2	55	25.5
3	48	22.2
4	33	15.3
5	28	13.0
Total	216	100.0

The average weight of students was 63.7 ± 14.2 kg, the height was 1.92 ± 3.63 m and the BMI was 22.8 ± 4.3. (Table 2).

**Table 2:** Anthropometric measurements of medical students (*n* = 216)

	Mean	Median	SD	Minimum	Maximum
Weight (kg)^a^	63.7	63.0	14.2	40.0	107.0
Height (m)^a^	1.92	1.67	3.63	1.49	1.94
BMI^a^	22.8	22.3	4.3	15.6	37.1
Waist circumference(cm)^a^	75.22	74.25	10.74	55.00	112
hip circumference(cm)^a^	96.99	95.75	11.23	71.00	152.00

^a^Not normally distributed based on Kolmogorov–Smirnov test and Shapiro–Wilk test

*SD* Standard Deviation

The prevalence of obesity among students was 5.1%. Morbid obesity and overweight 1.4 and 22.2% respectively. While 58% within normal range Table 3.

**Table 3:** Classification of participants according to BMI (*n* = 216)

BMI class	No	%
Under weight	28	13.0
Normal	126	58.3
Overweight	48	22.2
Obese	11	5.1
Morbid obese	3	1.4
Total	216	100.0

**Fig. 1:**
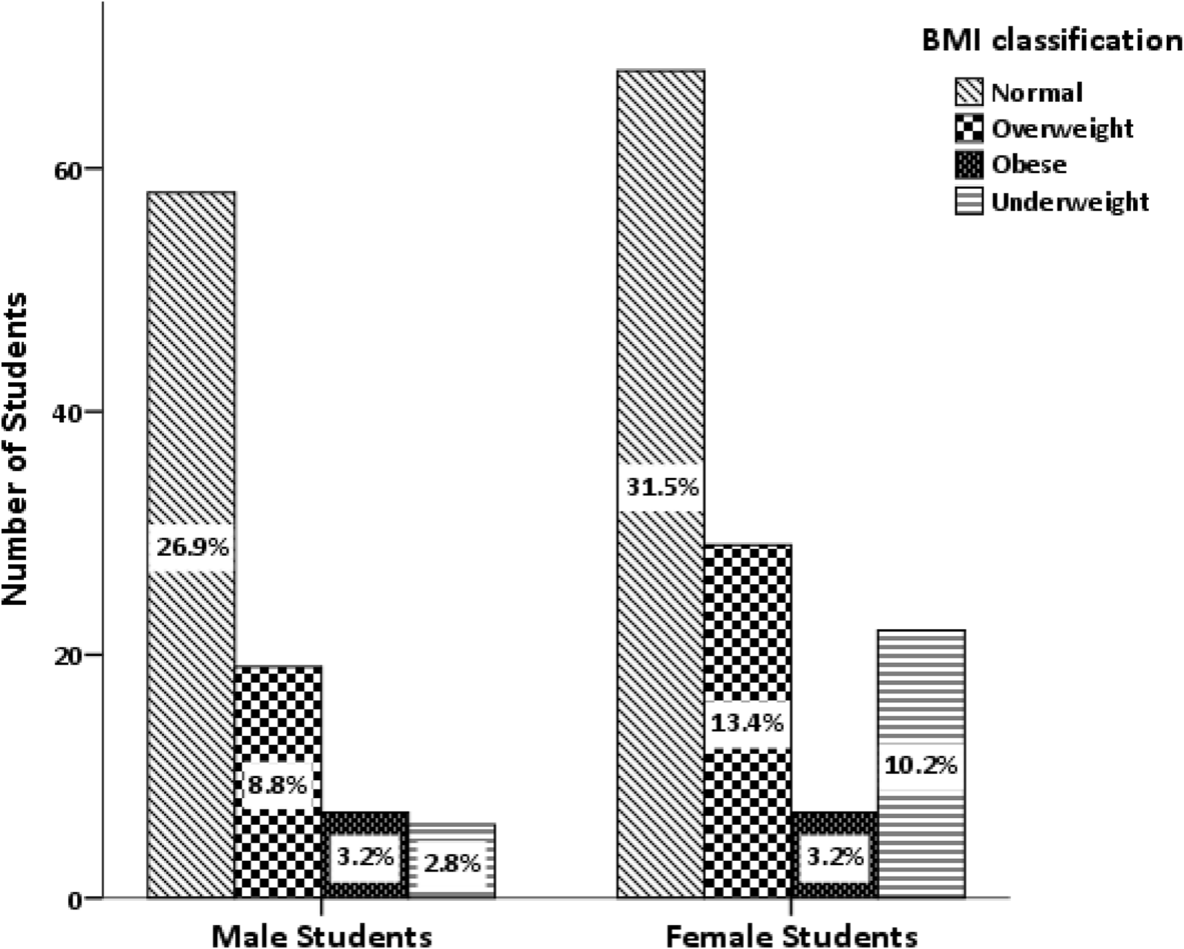
Association between gender and Body mass Index classification Chi-square value = 6.192 *P*-value of association test =0.103

Mann Whitney U test revealed No difference in BMI among males and females (*P* value = 0.513). Cross tabulation and chi-square test revealed no significant difference in BMI classification between males and females (*P* value = 0.103) (Fig. 1).

### Physical activity

Less than half of students (44.9%) had low activity level, 32.0% had moderate activity level and 23.1% had high Physical activity level. Table 4.

**Table 4:** Classification of students according to activity level (*n* = 216)

Physical activity level	No	%
Low	97	44.9
Moderate	69	32.0
High	50	23.1
Total	216	100.0

**Fig. 2:**
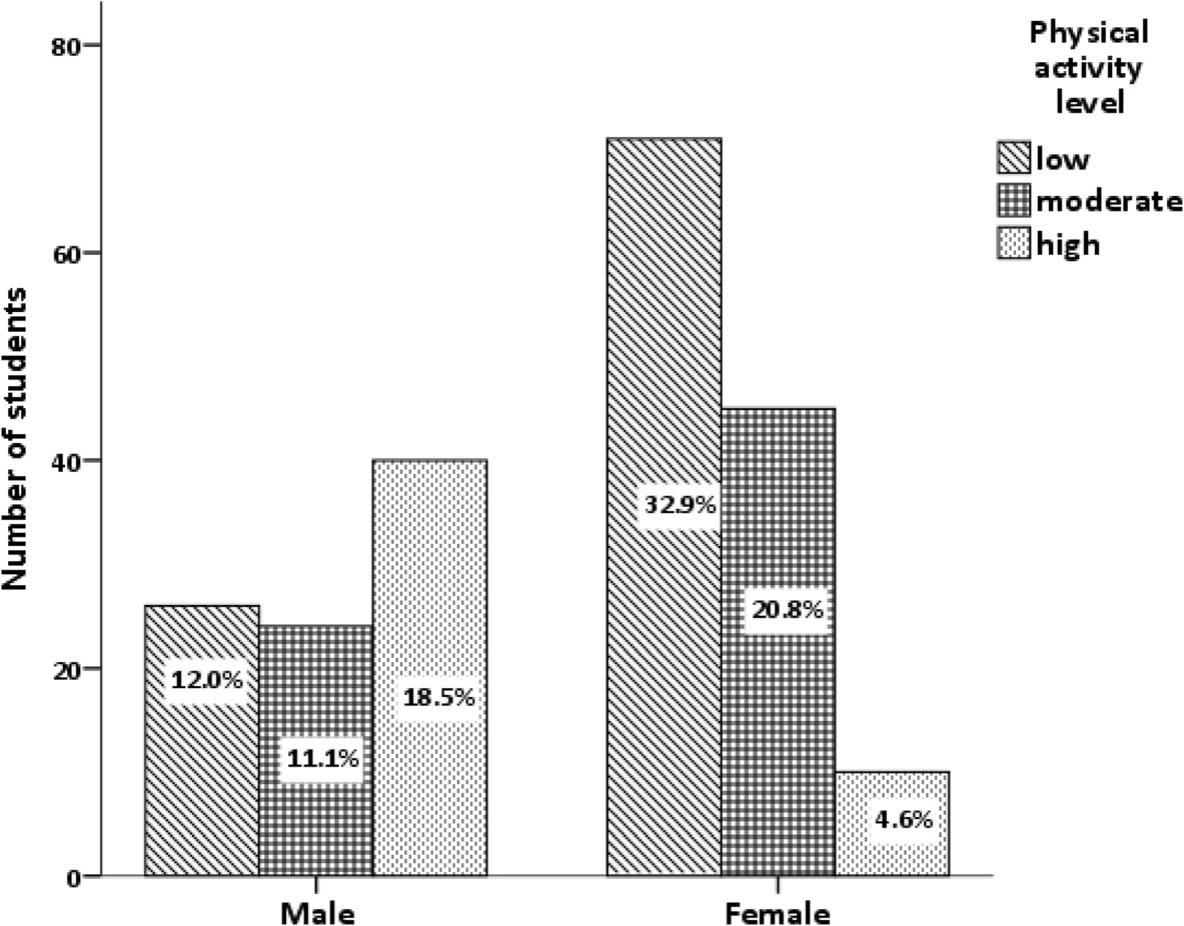
Association between gender and Physical activity Chi-square value = 40.4 *P*-value of association test < 0.0001

Cross tabulation and chi-square test revealed significant difference in physical activity level between males and females (*P* value< 0.0001) (Fig. 2).

**Fig. 3:**
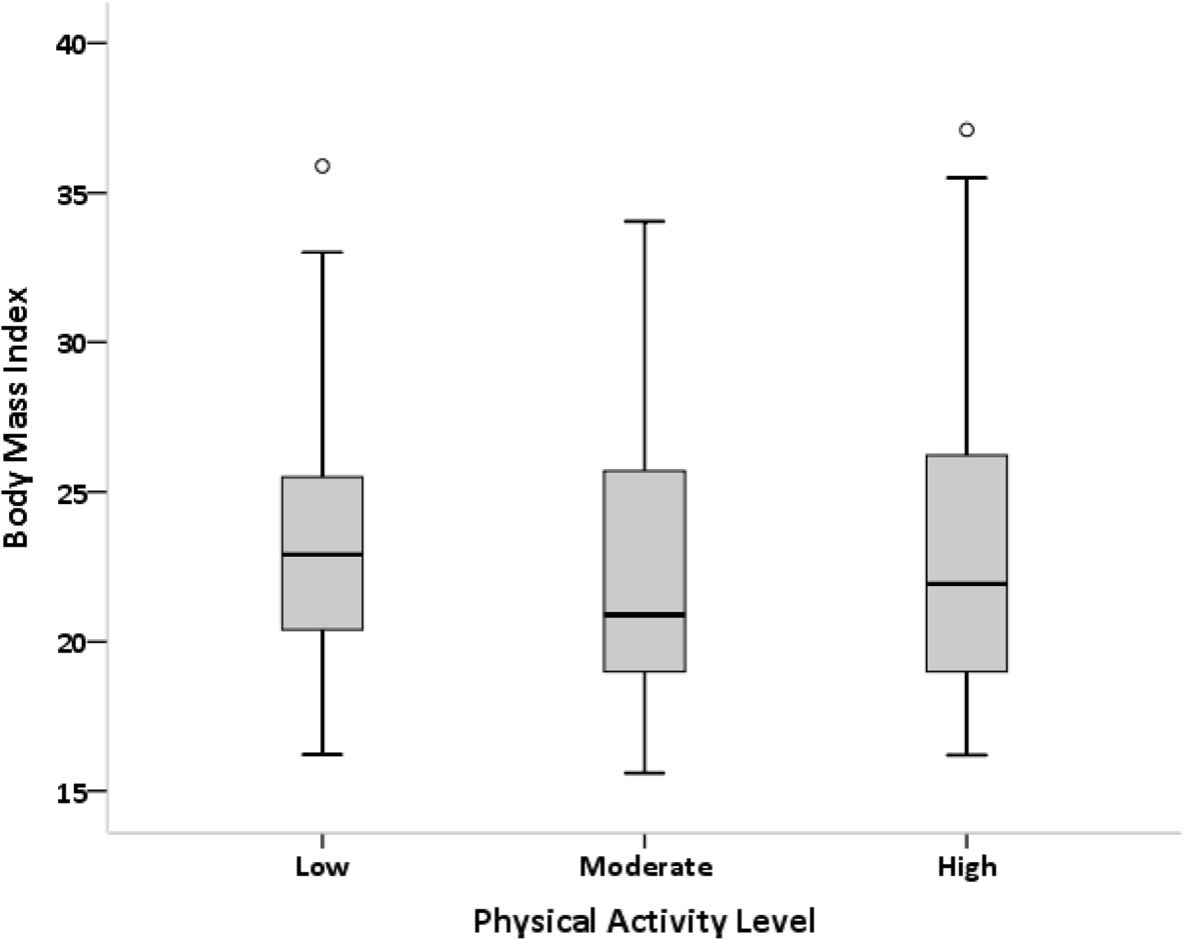
Relationship between physical activity level and BMI *P*-value =0.133

Kruskal-Wallis test showed no significant relationship between physical activity level and BMI (*P* value = 0.133) (Fig. 3).

### Eating behaviors

Near half of students were uncontrolled eaters (45.8%) followed by conscious restriction (28.7%) and then emotional eaters (25.5%). (Table 5).

**Table 5:** Distribution of participants according to eating behavior (n = 216)

Eating behaviour	NO	%
Uncontrolled eaters (UC)	99	45.8
Conscious restraints (CR)	62	28.7
Emotional eaters (EE)	55	25.5
Total	216	100.0

Kruskal-Wallis 1-way ANOVA test showed significant difference in BMI across the three groups of eating behaviors, (*P* value = 0.01).

## Discussion

The current study revealed that the prevalence of obesity among medical students at Al-Neelain University was 6.5%. This percentage is comparable previously reported among medical students from Sudanese University 9.2% [14]. However, the prevalence in the present context is still higher than the prevalence among other colleges in Sudan as the prevalence of obesity was 1.7% in non-medical colleges at Khartoum University [8] . The prevalence of obesity among Al-Neelain medical students showed in this study is less than that in the general population of Sudanese adults which was found to be 14.5 and 25.6% in Karai [15] and Jabra [16] districts respectively.

On the other hand, the prevalence of obesity found in this study is similar to neighboring countries but less than developed countries. In United Arab Emirates and Egypt the prevalence of obesity in medical students were 6.9 and 12.5% respectively [17, 18] . In Saudi Arabia, the prevalence of obesity among medical students at Imam Mohammed bin Saud Islamic university was 20% [19].At Kuwait university the prevalence of obesity among non-medical students was 19.8% [20]. A Study conducted at Crete School of Medicine in Greece found that the prevalence of overweight and obesity was 39.5% in male and 23.3% in female students [21]. The prevalence of obesity among medical students in Malaysia varied greatly as it was 2.8% or 5.2% in some colleges [22, 23] while it was 30% in other colleges [22]. In India, at Delhi University 3.4% of medical students were obese [24]. These discrepancies in results between these studies may be attributed to the differences in methods used to assess physical activity level and obesity.

This study revealed insignificant correlation between gender and prevalence of obesity. This could be explained since there was no gender difference in physical activity levels among students. This is in agreement with other studies in Sudan [25], Malaysia and India [26].However, other studies showed higher prevalence either among males [22, 27] or females [28].

It is well known that the practice of physical exercise can have important benefits in terms of preventive and therapeutic effects on health [29]. We assumed that medical students are aware of the importance of healthy lifestyles. Nevertheless, knowledge is only a part of expressing behaviors and providing health promotion. Also, there is no evidence to indicate that this knowledge translates into practice in terms of maintaining good health [30] .The present study showed that 44.4% of medical students had low physical activity level, 32% had moderate and only 23.1% had vigorous activity level. These values of physical activity are comparable to others studies among medical students worldwide. About two thirds of the medical students at Ain Shams university in Egypt used to practice exercise with 26.9% of these students practiced exercise for less than 2 h per week, while 33.9% of them for more than 2 h [18]. On the other hand, in Southern Thailand Approximately half (49.5%) of the medical students were physically active [31].

Our study showed significant difference in physical activity levels between male and female students. Insignificant relationship between gender and physical activity had been documented by previous studies [18, 32]. Although, other studies either disclosed higher level of physical activity among males [30, 31, 33] or females [34].

Our study revealed no relationship between physical activity level and BMI. This finding is in consistent with earlier studies which revealed no significant association between physical activity level and prevalence of obesity among medical students [18, 35, 36]. However, other studies showed either positive [30, 37] or negative relationship [38, 39].

The current study, distinguished between different eating patterns of eating behaviors, the uncontrolled eating, conscious restraints and emotional eating by using the TFEQ-r18 which had been used previously [40]. There was significant relationship between BMI and eating behaviors which is similar to previous work which revealed significant relationship between eating behavior and BMI [41].

The limitations of this study are it was conducted among one university and only among medical students. And we utilized only short IPAQ version. However, it is the first study, in Sudan, that used well-recognized and validated methods to assess physical activity level and eating behavior.

## Conclusion

In conclusion, low physical activity level and unhealthy eating behavior among medical study encourages implementation of health education programs about obesity and risk factors among medical students. Extracurricular physical sports and activates should be implemented by the administrations to encourage students to be more physically active in particular among female students.

Further studies are needed to detect determinants of obesity in non-medical students and in general population in aim to compare and to explore the possible mechanisms behind obesity among young adults.

## Abbreviations

BMI: Body Mass Index
CR: Cognitive restraint
EE: Emotional eating
IPAQ: International Physical activity questionnaire
TFEQ-R18: The three factor eating questionnaire revised 18
UE: Uncontrolled eating
